# THE FACES OF ELDER ABUSE: A CROSS-CULTURAL PERSPECTIVE

**DOI:** 10.1093/geroni/igac059.1120

**Published:** 2022-12-20

**Authors:** Pamela B Teaster Teaster, Georgia Anetzberger

## Abstract

This presentation gives a real world and compelling picture of the abuse of older adults, with authors representing three different World Health Regions (i.e., Regional Office for Africa, Regional Office for Southeast Asia, and the Regional Office for the Western Pacific) each locating the problem within an area’s historic and present societal treatment of older persons. An actual and emblematic case study of the abuse of an older adult will frame each presentation. Presenters will synthesize empirical data and research on the problem, explaining its usefulness and limitations as well as guiding frameworks utilized to address the problem, highlighting efforts of leading figures in each area or region who are addressing the problem and explaining existing policies and future initiatives to address the abuse of older adults. Dr. Eniola Cadmus will present perspectives on elder abuse in Nigeria. Drs. Noriko Tskudada and Asako Katsumata and will present perspectives from Japan. Drs. Farida Ejaz and Mala Shankardass will present perspectives from India. Dr. Georgia Anezberger will tie together the differences and commonalities within each region drawing upon a holistic and person-centered picture of the issue and problem of elder abuse.

